# Sub-second thermoplastic forming of bulk metallic glasses by ultrasonic beating

**DOI:** 10.1038/srep17844

**Published:** 2015-12-08

**Authors:** Jiang Ma, Xiong Liang, Xiaoyu Wu, Zhiyuan Liu, Feng Gong

**Affiliations:** 1 Key Laboratory of Advanced Manufacturing Technology for Mold & Die, College of Mechatronics and Control Engineering, Shenzhen University, Shenzhen 518060, PR China

## Abstract

The work proposed a novel thermoplastic forming approach–the ultrasonic beating forming (UBF) method for bulk metallic glasses (BMGs) in present work. The rapid forming approach can finish the thermoplastic forming of BMGs in less than one second, avoiding the time-dependent crystallization and oxidation to the most extent. Besides, the UBF is also proved to be competent in the fabrication of structures with the length scale ranging from macro scale to nano scale. Our results propose a novel route for the thermoplastic forming of BMGs and have promising applications in the rapid fabrication of macro to nano scale products and devices.

Since their discovery in 1960s^1^, the unique properties of metallic glasses, such as high strength, high specific strength, large elastic strain limit, and excellent wear and corrosion resistances along with other remarkable engineering properties have made these materials of significant interest for science and industry^1^,^2^,^3^,^4^,^5^,^6^,^7^,^8^,^9^,^10^,^11^,^12^. One of the most intriguing features of metallic glasses is their ability to be thermoplastically formed like plastics above the glass transition temperature *T*_*g*_. This “thermoplastic forming” ability was widely perceived as finally bridging the gap between the manufacturing of metals and that of plastics^13^,^14^,^15^,^16^,^17^,^18^. Benefiting from the ability, bulk metallic glasses (BMGs) can be formed into parts of complex surface structures with well-defined geometry and shape on length scales ranging from macro, micro, to even nano^19^,^20^,^21^,^22^,^23^,^24^, which is almost impossible if we use the traditional mechanical machining methods due to their lack of plasticity at ambient temperature. Based on this property, researchers have exploited different conventional thermoplastic techniques such as hot embossing^25^,^26^, blow molding^27^ and injection molding^28^ *et al*. to fabricate various structures and products using BMGs. It is widely accepted that thermoplastic forming is one of the most promising fields for the application of BMGs^11^,^13^,^29^.

The thermoplastic forming of BMGs always involves heat treatment. As a metastable material, BMGs tends to transform from amorphous to crystalline state under certain temperature. The evolution of this trend can be summarized in a temperature-time-transformation (TTT) diagram, as shown in Fig. 1. One can see that at a higher temperature *T*_*processing*_ in the supercooled liquid region (SLR, a temperature window between glass transition temperature *T*_*g*_ and crystallization temperature *T*_*x*_) of a BMG former, a less processing time *t*_*processing*_ is left to us to handle. As illustrated by the red lines in Fig. 1, if a BMG former is treated by the temperature routine without crossing the crystalline region such as I and II, it keeps the amorphous nature after the thermoplastic processing, otherwise, it will get crystallized like III. Therefore, the main challenge for thermoplastic forming of BMGs turns out to be how to avoid crystallization. Obviously, the efficient strategy would be shortening the processing time *t*_*processing*_ as possible as one can. Unfortunately, the time window *t*_*processing*_ is generally quite limited for most BMG formers^30^. This greatly limited the potential applications of thermoplastic forming of BMGs, therefore, a novel thermoplastic method to overcome these limitations is widely expected.

Previous work on the thermoplastic forming of BMGs usually needed separated steps for heating, force applying, and quenching^13^,^16^,^17^,^24^,^26^, i. e., the BMG sample was firstly heated to a desired temperature and held for a period of time to attain thermal equilibrium, then, the required force was applied to drive the sample to flow. Following this procedure, much prime time was inevitably wasted, leading the BMGs at the risk of being crystallized and oxidized. So, the question was then raised: Was there any possibility that these steps could work simultaneously ?

As we know, if one pounds a metal surface rapidly and repeatedly by a hammer, it can be found that the place where the hammer strikes the metal warms up due to friction. Now imagine pounding that metal thousands of times per second, the frictional heat generated would raise the temperature significantly in a very short time^31^. Inspired by this phenomenon, we could develop the ultrasonic beating forming (UBF) of BMGs. As illustrated in Fig. 2(a), the BMG sample was repeatedly beaten under certain force *F* by a cylinder ultrasonic indenter (with a diameter of 5 mm) at 20000 times per second for a very short period of time *t*, causing the BMG sample to get hot and soft, resulting in thermoplastically formed. During the above UBF process, heating and forming work simultaneously in an instant, after that, the beating stops and the heat transfers away immediately from the BMG sample when the forming process finishes. Actually, similar ideas were used to weld or bond bulk metallic glasses together^32^,^33^. Apparently, compared with conventional thermoplastic forming appliance, no special heating devices are required in present approach, and the main difference between UBF and conventional thermoplastic forming methods would be the needed processing time, which could be visually reflected by the routine I and II in Fig. 1. The UBF can greatly shorten the required processing time (tens of times less than conventional methods), making a lot of sense of avoiding crystallization and oxidation in the thermoplastic forming of BMGs.

## Results and Discussion

Figure 3(a) shows the photographs of BMG samples before and after the UBF treatment, the details of sample B and C are revealed in Fig. 3(b,c). From the deformed BMG sample B and C, we can see the typical thermoplastic forming took place during the UBF process, and the deformation concentrated in a circular zone with a diameter of 5 mm, just in the punching area of the ultrasonic indenter. Amazingly, the BMG plate could be even trimmed cleanly by the indenter, like blanking process in traditional machining. As it is shown in Fig. 3(c), the circular cutting edge is very clean, this is almost impossible to finish by conventional mechanical machining techniques for BMGs. In addition, a very thin gauge thermocouple wire coupling with a high rate of data acquisition was used to detect the temperature change during the UBF. From the obtained temperature curve (as presented in Fig. 3(f)), it can be seen that the temperature of the Zr_35_Ti_30_Cu_8.25_Be_26.75_ BMG specimen ramps up to 377 °C in a very short time. The maximum temperature in the curve is higher than the transition temperature *T*_*g*_, yet lower than the crystallization temperature *T*_*x*_. Therefore, the sample kept the amorphous nature after being processed, the x-ray diffraction (XRD) pattern and differential scanning calorimetry (DSC) curve of the deformed area are presented in Fig. 3(d,e). The invariance of structure is the basic requirement of thermoplastic forming, and it could be guaranteed by the short time that is needed through UBF.

According to the heat equation, 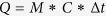 where *m*, *C* and Δ*t* are the mass, specific heat capacity and temperature raise of an object when given a heat *Q*. In present case, the values of *m*, *C* and Δ*t* are 0.1 g, 0.417 J/(g · K) and 305 °C, respectively^34^,^35^. Therefore, the heat quantity needed to drive the BMG sample into its SLR would be about 12.72 *J*. On the other hand, the work done by the ultrasonic indenter could be calculated by the following equation:


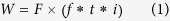


where *F* = 424 N is the applying force of the ultrasonic indenter, *f* = 20000, *t* = 0.5 s and *i* = 5 μm are the beating frequency, forming time and displacement per beating of the ultrasonic indenter. By substituting the above values into Eq. (1), we obtain *W* = 21 J. Here, we can see the work done by the ultrasonic indenter *W* is greater than the required heat quantity *Q*, indicating the ultrasonic beating could provide enough energy for the BMGs to get soft. However, it does not bring in crystallization to BMGs, or rather, the energy can be divided into two parts: one is used to heat up the BMG sample into its SLR and the extra one is dissipated by the deformation after it gets soft.

**Figure 1:**
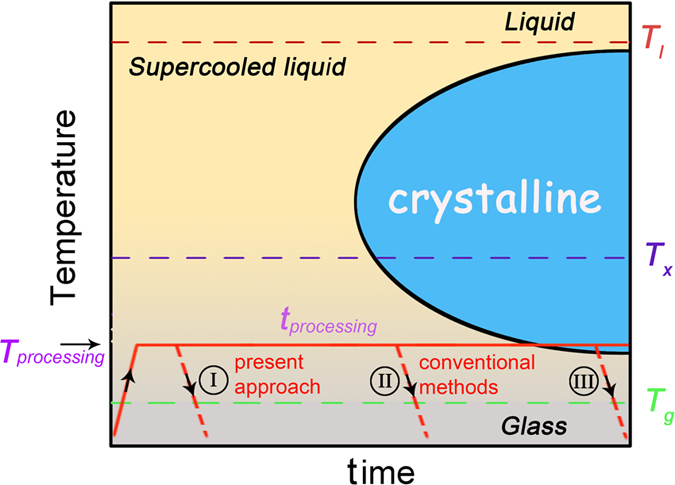
Schematic TTT diagram for a metallic glass former. I, II and III represent different temperature routines.

The unique thermoplastic forming ability of BMGs may have promising applications in many fields especially in the small-scale devices^13^,^14^,^21^,^24^. Here, the key issue would be how to avoid oxidation and crystallization during the essential high temperature treatment^13^. As two dynamic processes, oxidation and crystallization are strongly effected by the holding time at high temperature^36^,^37^, i. e., the two adverse factors for thermoplastic forming of BMGs can be weakened to the most extent if the processing time (See Fig. 1) could be as short as possible. For conventional thermoplastic forming strategy, BMGs need to stay at high temperature for at least several seconds even minutes^38^, which would bring them into the danger of getting oxidized or crystallized. However, the time needed for UBF is very short (less than one second), thus, the BMGs are much safer through UBF than conventional thermoplastic methods in terms of oxidation and crystallization. Benefiting from this advantage, the UBF may be used to form some BMG systems that were considered to be not suitable for thermoplastic forming by conventional techniques.

**Figure 2:**
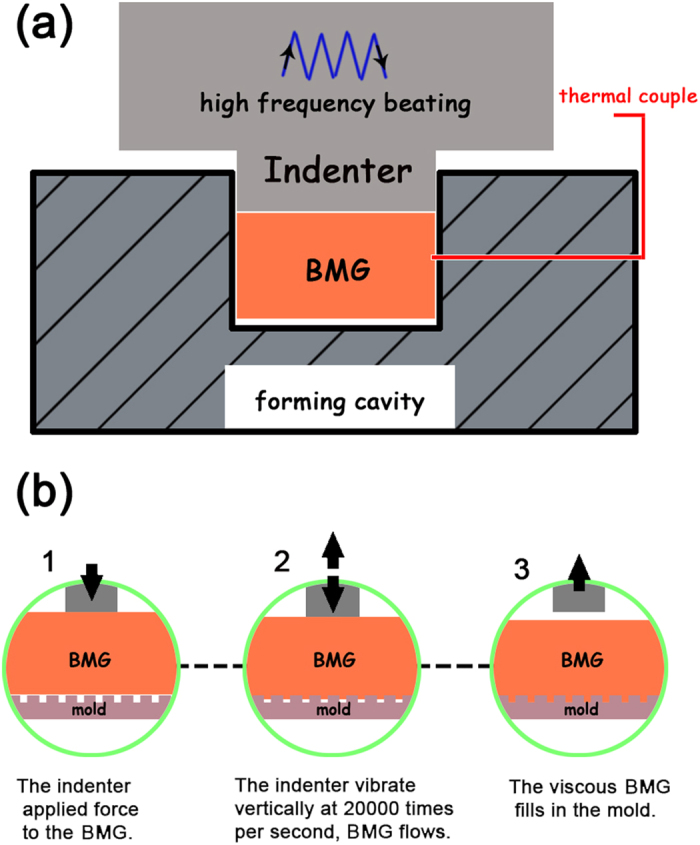
(**a**) Schematic diagram of the ultrasonic beating forming (UBF) setup. (**b**) Illustration of the UBF process in molds.

**Figure 3:**
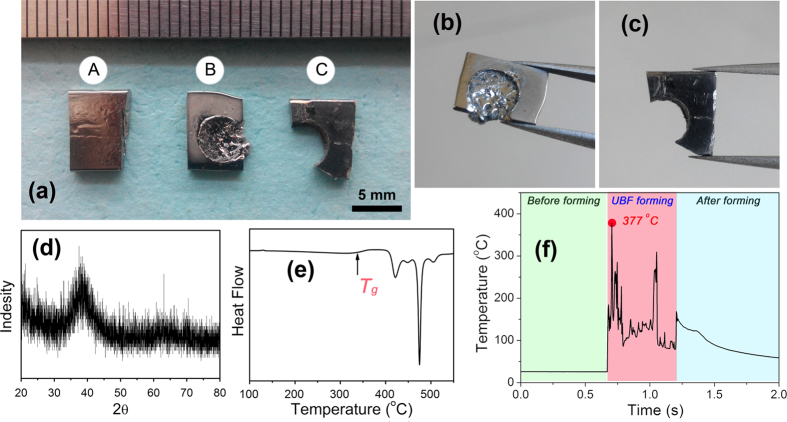
(**a**) The photographs of BMG samples before and after the UBF treatment, the details of sample B and C are revealed in (**b**,**c**), respectively. (**d,e**) present the XRD pattern and DSC curve of the BMG sample after UBF. (**f**) shows the temperature curve of the BMG specimen during UBF process.

It is believed that the thermoplastic forming of BMGs makes them suitable and ideal for the fabrication of products and devices with the length scale ranging from tens of centimeters down to several nanometers^13^. To validate if the sub-second UBF is competent for this kind of work, structures from macro scale to micro and even nano scale were tried to fabricate on the surface of BMGs through this method, as illustrated in Fig. 2(b). The ultrasonic indenter applied force to the BMG sample and vibrate vertically at a frequency of 20000 times per second, causing the BMG sample to get soft and fill into the mold, and the whole forming process lasts for only less than one second (typically about 0.5 s). During the macro scale fabrication, the molds were made of stainless steel, and in the cases of micro and nano scale fabrication, the molds were substituted by the silicon molds and the anodic aluminum oxide (AAO) templates, respectively.

**Figure 4:**
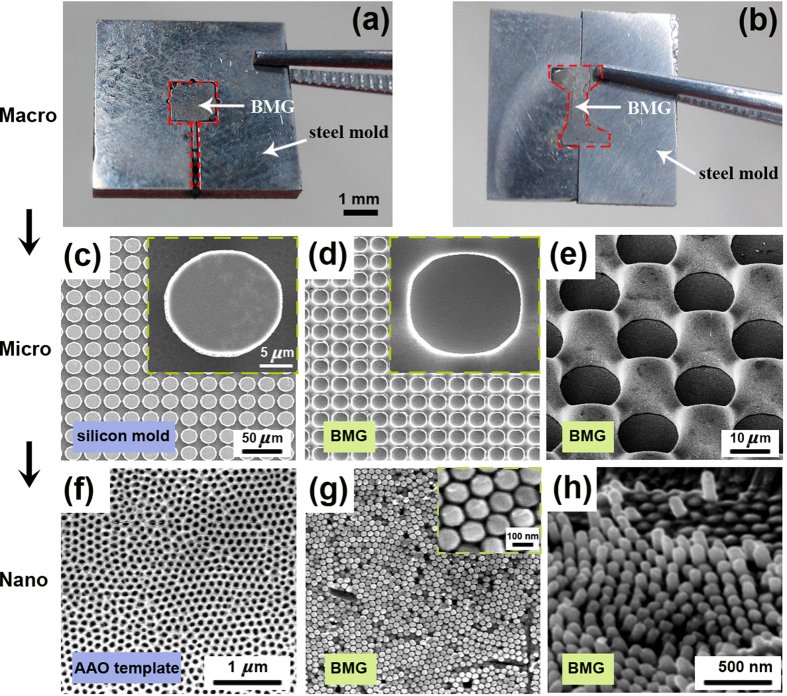
Structures from macro scale to nano scale on BMG surfaces fabricated by the UBF. (**a,b**) show BMGs fill into rectangular hole and the dog-bone shaped hole; (**c**), (**d**,**e**) show the SEM images of the silicon molds and corresponding BMG sample after UBF, the insets present the details. (**f**), (**g**,**h**) reveal the nano scale morphology of the AAO template and the as-fabricated BMG surface. (**e,h**) are the angled views of (**d,g**). (**a**–**d**,**f**,**g**) share the same scale bar, respectively.

Figure 4(a,b) show the photos of BMGs which were squeezed into the macro mold cavities. For the convenience of comparison, two different steel mold cavities were fabricated: the rectangular hole and the dog-bone shaped hole. It can be seen that the BMG fully fills into the mold cavities through the UBF and the as-fabricated BMG parts keep their original metallic luster, revealing no oxidation occurs to them. These results indicate the UBF could be competent for the rapid fabrication of precise BMG macro parts and even mechanical testing samples. In addition to the macro forming, the UBF can also be used to fabricate micro or even nano structures on the surface of BMG. Figure 4(c,d) present the scanning electron microscope (SEM) images of the micro scale patterns on the surface of the silicon molds and the corresponding BMG surfaces, respectively. The insets in Fig. 4(c,d) show the details of them. One can see from the SEM observation that the diameter of the periodic micro cylinders on the silicon mold is 20.56 μm and the diameter of the micro holes on the replicated BMG is 20.34 μm, with only a small dimension mismatch of about 1%, indicating that the UBF method is effective and precise to fabricate the controllable micro structures on the surface of BMGs. The angled view of micro-structured BMG surface after UBF is shown in Fig. 4(e), one can clearly see the circular cavities and the protrusions around them in a three-dimensional sense. Besides above, the nano structures were also successfully fabricated by the proposed UBF approach. Figure 4(f) shows the SEM morphology of the anodic aluminum oxide (AAO) template that was used as molds during the nano structure fabrication. It can be seen the regular nano-scale pores with a mean diameter of ~80–100 nm are evenly distributed on the AAO surface. The SEM image of the as-fabricated BMG surface after UBF is presented in Fig. 4(g), and it can be seen that distinct nano structures were produced. The inset in Fig. 4(g) clearly shows the dimension of nano pillars is just in coincidence with the AAO templates. Furthermore, the angled view in Fig. 4(h) reveals the nano structure morphology more visually. These results above indicate that the UBF is effective for BMGs in the fabrication of macro-scale structures or parts as well as micro and even nano patterns, exhibiting huge advantages compared with conventional thermoplastic forming methods in fighting against the crystallization and oxidation.

## Conclusion

In summary, the developed UBF method in present research provides a novel sub-second thermoplastic forming approach for BMGs. The rapid forming combines temperature ramping and force giving processes together, making it practical to finish the thermoplastic forming of BMGs in less than one second. Benefiting from this, the risk of being crystallized and oxidized can be greatly reduced. Therefore, the proposed UBF could provide a safe environment for the thermoplastic forming of BMGs. Our results open a new route for the thermoplastic forming of BMGs and have promising applications in the rapid fabrication of macro to nano products and devices on the surface of metallic materials.

## Methods

### Materials

The Zr_35_Ti_30_Cu_8.25_Be_26.75_ BMG plate samples were prepared from a master alloy with nominal composition of Zr 35 at.%, Ti 30 at.%, Cu 8.25 at.%, and Be 26.75 at.% by conventional water cooled copper mould casting process. For the convenience of UBF, the plate was cut into a size of 8 mm × 5 mm × 1 mm and then the surface was polished by the abrasive paper and polishing machine.

### Ultrasonic beating forming (UBF)

To conduct the ultrasonic beating forming (UBF), a strong punch is needed to beat the surface of bulk metallic glasses repeatedly at a high frequency. The ultrasonic indenter plays such a role. It is made of cemented carbide (TC4 titanium alloy in present work) and bonded together with a converter or piezoelectric transducer which converts the electrical signal into a mechanical vibration. Therefore, the indenter could vibrate very fast (*frequency* = 20000 HZ in present work) and then apply the mechanical vibration to the BMG specimens to be formed. The Zr-based BMG plate sample was placed under the ultrasonic indenter, then a force of 424 N was applied and the indenter started to vibrate under a frequency of 20000 times per second. The beating time lasted for 0.5 s.

### Characterization

The amorphous nature of the Zr-based BMG samples was ascertained by x-ray diffraction (XRD) with Cu K_α_ radiation and differential scanning calorimetry (DSC; Perkin–Elmer DSC-7) at a heating rate of 10 K/min. The surface morphologies were collected on an Philips XL30 scanning electron microscope (SEM) instrument.

## Additional Information

**How to cite this article**: Ma, J. *et al*. Sub-second thermoplastic forming of bulk metallic glasses by ultrasonic beating. *Sci. Rep*. **5**, 17844; doi: 10.1038/srep17844 (2015).
